# RNA-Based Stable Isotope Probing Suggests* Allobaculum* spp. as Particularly Active Glucose Assimilators in a Complex Murine Microbiota Cultured In Vitro

**DOI:** 10.1155/2017/1829685

**Published:** 2017-02-16

**Authors:** Elena Herrmann, Wayne Young, Douglas Rosendale, Verena Reichert-Grimm, Christian U. Riedel, Ralf Conrad, Markus Egert

**Affiliations:** ^1^Institute of Precision Medicine, Faculty of Medical & Life Sciences, Furtwangen University, 78054 Villingen-Schwenningen, Germany; ^2^AgResearch Ltd, Grasslands Research Centre, Palmerston North 4442, New Zealand; ^3^The New Zealand Institute for Plant & Food Research Ltd, Palmerston North 4442, New Zealand; ^4^Institute of Microbiology and Biotechnology, University of Ulm, 89069 Ulm, Germany; ^5^Department of Biogeochemistry, Max Planck Institute for Terrestrial Microbiology, 35043 Marburg, Germany

## Abstract

RNA-based stable isotope probing (RNA-SIP) and metabolic profiling were used to detect actively glucose-consuming bacteria in a complex microbial community obtained from a murine model system. A faeces-derived microbiota was incubated under anaerobic conditions for 0, 2, and 4 h with 40 mM [U^13^C]glucose. Isopycnic density gradient ultracentrifugation and fractionation of isolated RNA into labeled and unlabeled fractions followed by 16S rRNA sequencing showed a quick adaptation of the bacterial community in response to the added sugar, which was dominated by unclassified Lachnospiraceae species. Inspection of distinct fractions of isotope-labeled RNA revealed* Allobaculum* spp. as particularly active glucose utilizers in the system, as the corresponding RNA showed significantly higher proportions among the labeled RNA. With time, the labeled sugar was used by a wider spectrum of faecal bacteria. Metabolic profiling indicated rapid fermentation of [U^13^C]glucose, with lactate, acetate, and propionate being the principal ^13^C-labeled fermentation products, and suggested that “cross-feeding” occurred in the system. RNA-SIP combined with metabolic profiling of ^13^C-labeled products allowed insights into the microbial assimilation of a general model substrate, demonstrating the appropriateness of this technology to study assimilation processes of nutritionally more relevant substrates, for example, prebiotic carbohydrates, in the gut microbiota of mice as a model system.

## 1. Introduction

It is well documented that symbiotic gut inhabitants are required for maintaining host health and well-being [1, 2], as they greatly influence several host functions, not only those in the intestinal system [3–8]. Despite the rapid development of next generation sequencing technologies, which provide deep insight into the structure of the human microbiome [1], metabolically active populations in this complex community are still poorly described and incompletely understood. Hence, there is still a large knowledge gap of the in situ functionality of the gut microbiota and their metabolic capacities at an overall population level. To obtain a more complete picture and a better understanding of functional features of the gut microbiome in health and disease, we need to determine the in situ metabolic function of individual species within this complex microbial community.

Ingestion of prebiotic carbohydrates to boost health-promoting intestinal fermentation by selective enrichment and/or stimulation of the activity of commensal microorganisms that contribute to the well-being of their host is a viable strategy to improve host health through the benefits of microbial metabolism [9–11]. In particular short chain fatty acids (SCFA), such as acetate, propionate, and butyrate produced during microbial fermentation, have attracted attention in this regard. For instance, butyrate represents the main energy source for colonocytes [12], and anti-inflammatory and immune-modulating effects of butyrate have been observed [7, 13, 14]. However, knowledge about which microbial populations are stimulated by which carbohydrate under the in situ conditions of the human gastrointestinal (GI) tract or other intestinal (model) systems is limited. The ability to link specific nutrient metabolizing processes to distinct groups of intestinal microorganisms would greatly expand the understanding of the dynamics of prebiotic carbohydrate utilization and their fate in the large bowel.

The stable isotope probing (SIP) technology [15] is a means of identifying microorganisms associated with various assimilation and fermentation processes of highly isotope-enriched compounds in environmental samples [16–19]. RNA-SIP [20] offers the advantage of a rapid incorporation of the stable isotope (e.g., ^13^C) into the RNA by metabolically active microorganisms because of a high RNA synthesis rate [17]. This ^13^C-enriched RNA (“heavy” RNA) can then be separated from unlabeled ^12^C background RNA (“light” RNA) using isopycnic density gradient centrifugation [21, 22]. Further molecular characterization of the 16S rRNA in the fractionated gradients links bacterial identity to metabolic activity. This technology has been applied to a few gut microbiota before, studying the utilization of simple and complex sugars [23–26], and it has been proven to provide an effective link between the identity of microorganisms and the metabolic assimilation of a particular substrate in situ. Otherwise, this information can only be indirectly inferred from traditional enrichment type studies using nonlabeled substrates. In addition, high pressure liquid chromatography coupled to isotope ratio mass spectrometry (HPLC-IRMS) is a valuable technique for tracking the ^13^C-label down into various metabolic products including volatile fatty acids [27–29]. Their quantification and isotopic composition provide additional insight into the functioning of microbial communities, as distinct populations can be linked to specific metabolic pathways [30, 31].

Animal models represent valuable and relatively easy accessible tools in gut microbiota research [32, 33]. In particular in vivo studies are important for studying basic aspects of digestion, such as the microbial assimilation of prebiotic carbohydrates, and help to unravel the functionality of the intestinal microbiota and to better understand its role in health and disease. Here, to the best of our knowledge, RNA-SIP was used for the first time in an intestinal environment of mice origin. In combination with metabolic profiling, bacteria being able to assimilate carbohydrates within a complex murine faecal microbiota cultured in vitro were identified. Using [U^13^C]glucose as a simple and quick fermentable model substrate, active glucose fermenters in the system were detected already after 2 h of incubation. The detected ^13^C-labeled metabolic products corroborated the RNA-based sequencing results. Our study represents a “proof of principle” study establishing the use of RNA-SIP technology to interrogate samples of mice origin, which, to the best of our knowledge, has not been done before. Using this study as a starting point, future studies will address utilization of more complex carbohydrates, such as resistant starch (RS), by communities of mice origin in vivo to identify prebiotics assimilating bacteria directly in the intestinal environment.

## 2. Material and Methods

### 2.1. Collection and Cultivation of Murine Faecal Samples

C57BL/6J mice were raised at the animal facility at the University of Ulm under specific pathogen-free (SPF) conditions, receiving a standard laboratory diet and water ad libitum. Fresh faecal pellets were randomly collected from 12 healthy animals within 4 h of excretion. The pellets of all animals were finally pooled and subsequently homogenized in M9 minimal medium [34] supplemented with thiamin (2 mg/L) and Casamino Acids (1 g/L), lacking glucose at this stage, to obtain a 15% (w/v) faecal slurry. Slurry material (1 mL for each treatment) was mixed 1 : 1 with M9 minimal medium containing 80 mM of [U^13^C]glucose (≥99 atom%) (Sigma-Aldrich GmbH, Steinheim, Germany), yielding a final concentration of 40 mM glucose and 7.5% (w/v) of faecal material. Samples were incubated in individual 15 mL reaction tubes at 37°C for 0 h (control), 2 h, or 4 h under anaerobic conditions, which were obtained by using airtight jars and AnaeroGen sachets (Merck, Darmstadt, Germany) resulting in a total of three different incubation treatments. Prior to use, the M9 minimal medium was filter sterilized, deoxygenated, and prewarmed to 37°C.

### 2.2. RNA Extraction, Isopycnic Centrifugation, Gradient Fractionation, and Quantification

After incubation, microbial cells from each incubation were harvested by centrifugation for 20 min at 4°C and 3220 ×g. Total RNA was extracted from 1 mL of each sample using the PowerMicrobiome RNA Isolation Kit following the manufacturer's instructions (MO Bio Laboratories Inc., Carlsbad, USA). Residual genomic DNA was removed by incubating the RNA (100 *μ*L) for 1 h at 37°C with 10 *μ*L RNase-free DNase I (10 U/*μ*L; Roche, Mannheim, Germany), 15 *μ*L of 10x DNase I buffer (400 mM Tris-HCl, 100 mM NaCl, 60 mM MgCl_2_, 10 mM CaCl_2_, pH 7.9), and 25 *μ*L of nuclease-free water. Further purification of the RNA samples was performed using Qiagen RNeasy spin columns (Qiagen, Hilden, Germany) according to the manufacturer's instructions. Subsequently, the RNA samples were quantified using a P360 Nanophotometer (Implen GmbH, Munich, Germany) and the absence of genomic DNA was confirmed by the absence of PCR products after amplification of 16S rRNA genes with universal primers 27F/907R [35, 36].

Gradient ultracentrifugation to density-resolve the extracted RNA was performed following the method used by Egert and colleagues [23] with some adaptations. The centrifugation medium contained cesium trifluoroacetate (CsTFA) stock solution (6.66 mL; 2 g/mL, GE Healthcare, Freiburg, Germany), gradient buffer (1.455 mL; [21]) loaded with ~700 ng RNA, and 0.285 mL formamide (Roth, Karlsruhe, Germany). Subsequently, the density of the solution was checked using an AR200 digital refractometer (Reichert, Depew, NY, USA) and, if necessary, was adjusted to a refractive index of 1.3724 ± 0.0001, corresponding to a buoyant density (BD) of ~1.793 g/mL, by adding CsTFA stock solution or gradient buffer. Fraction density and refractive index were correlated by means of a previously established calibration curve. Next, the gradient solution was added to 8 mL Quick-Seal Polypropylene tubes (BeckmanCoulter Inc., Krefeld, Germany) and spun at 20°C and ~123,100 ×g (45,000 rpm) for 67 h using an MLN-80 rotor [37] in an Optima MAX-XP bench-top ultracentrifuge (both BeckmanCoulter). After ultracentrifugation, gradients were fractionated into 16 equal fractions (~0.5 mL) by displacement with water from the top of the tube under a consistent flow rate of 1 mL/min using a syringe pump (World Precision Instruments, Berlin, Germany). An aliquot of 75 *μ*L of each harvested fraction was used to measure density using the refractometer. RNA from these fractions was then precipitated by adding one volume of ice-cold isopropanol, washed with 150 *μ*L ice-cold 70% ethanol, briefly air-dried, and finally redissolved in 20 *μ*L of nuclease-free water. The concentration of RNA in each fraction was determined using a RiboGreen low range assay (Life Technologies GmbH, Darmstadt, Germany) on a microplate reader (Tecan Group Ltd, Männedorf, Switzerland) and a total assay volume of 200 *μ*L according to the manufacturer's instructions.

### 2.3. Reverse-Transcription, 16S rRNA Amplicon Library Construction, and Sequencing

Ten *μ*L of RNA from each gradient fraction was reverse-transcribed to cDNA using the SuperScript VILO cDNA Synthesis Kit (Life Technologies GmbH, Darmstadt, Germany) following the manufacturer's protocol.

To reveal the microbial community structure represented in the cDNA from the different density gradient fractions, 16S rRNA gene amplicon libraries of the V3 and V4 regions of the bacterial 16S rRNA gene were prepared according to Illumina's 16S Metagenomic sequencing library preparation protocol [38] with minor modifications. In brief, for PCR amplification of the region of interest, the 16 rRNA gene specific primers forward S-D-Bact-0341-b-S-17 (5′-CCTACGGGNGGCWGCAG-3′) and reverse S-D-Bact-0785-a-A-21, (5′-GACTACHVGGGTATCTAATCC-3′) were used [39], added to an overhang adaptor sequence tail (TCGTCGGCAGCGTCAGATGTGTATAAGAGACAG and GTCTCGTGGGCTCGGAGATGTGTATAAGAGACAG), respectively. The PCR amplification mixture with a final volume of 50 *μ*L consisted of 0.5 *μ*L of each primer (50 *μ*M), 1 *μ*L of dNTP-Mix (10 mM, of each), 1 *μ*L of BSA (20 mg/mL), 5 *μ*L of 10x DreamTaq buffer (including 20 mM MgCl_2_), 0.25 *μ*L of DreamTaq DNA-Polymerase (5 U/*μ*L), 39.75 of *μ*L nuclease-free water, and 2 *μ*L of cDNA template. PCR reactions were performed in a T100 Thermal Cycler (Bio-Rad Laboratories, Munich, Germany) using the following profile: 3 min at 95°C for initial denaturation, 25 cycles of 30 s at 95°C for denaturation, 30 s at 55°C for primer annealing, and 60 s at 72°C for elongation, followed by a final elongation step for 5 min at 72°C, yielding a PCR product of ~550 bp. Water-template control reactions were included for each batch. PCR products were verified by standard agarose gel electrophoresis. Aliquots (22 *μ*L) of PCR product were subsequently purified with Agencourt AMPure XP beads (BeckmanCoulter Inc.) according to the Illumina library preparation protocol [38]. Subsequently, a second PCR step was performed to anneal unique dual-index barcodes with Illumina sequencing adaptors to the amplicon target using the following reaction mixture in a total volume of 50 *μ*L: 5 *μ*L of Nextera XT Index 1 primer, 5 *μ*L of Nextera XT Index 2 primer (both from Nextera XT index kit; Illumina, Eindhoven, Netherlands), 5 *μ*L of PCR product, 1 *μ*L of dNTP-Mix (10 mM, each), 5 *μ*L of 10x DreamTaq buffer (including 20 mM MgCl_2_), 0.25 *μ*L of DreamTaq DNA-Polymerase (5 U/*μ*L), and 28.75 *μ*L of nuclease-free water. PCR was performed on a T100 Thermal Cycler (Bio-Rad Laboratories) using the following program: 3 min at 95°C for initial denaturation, 8 cycles of 30 s at 95°C for denaturation, 30 s at 55°C for annealing, and 30 s at 72°C for elongation and a final elongation period for 5 min at 72°C. Afterwards, the libraries were subjected to bead-purification using Agencourt AMPure XP beads (BeckmanCoulter Inc.) according to the Illumina protocol [38]. Prior to fluorometric quantification using the Qubit dsDNA HS assay kit (Life Technologies) and subsequent normalization to a final concentration of 4 nM, the quality of each library (size ~630 bp) was assessed using a Bioanalyzer DNA 1000 chip (Agilent Technologies GmbH, Waldbronn, Germany). Libraries were finally pooled in equivalent quantities and sequenced in duplicate on an Illumina MiSeq platform (Illumina) in a final concentration of 5.5 pM with 15% control phiX added using the MiSeq Reagent Kit v3 in a 600-cycle (2 × 300 bp) format (Illumina) following the manufacturer's instructions.

### 2.4. Sequencing Analysis and Statistics

The sequencing data were processed with QIIME 1.8 [40]. Overlapping paired-end Illumina fastq files were merged using the join_paired_ends.py script with default settings. Assembled sequences were quality filtered using a *Q*30 base call accuracy cut-off and allocated to their respective samples according to their unique barcode sequence. The demultiplexed sequences were then chimera checked using the USEARCH method against the Greengenes alignment (release GG_13_8). Sequences identified as chimeric were removed from subsequent analyses. Sequence reads were clustered into operational taxonomic units (OTUs) at 97% or greater similarity using the USEARCH method [41]. Representative sequences were aligned with PyNAST against the Greengenes database (release GG_13_8) and assigned taxonomies using the Ribosomal Database Project (RDP) classifier [42]. Alpha and beta diversity analyses were performed using the core_diversity_analyses.py script in QIIME 1.8. Alpha diversity was calculated through the phylogenetic metric PD_whole_tree (Faith's phylogenetic diversity estimate) using the value of the minimum number of reads (1008) across 10 iterations. Beta diversity was visualized using principal coordinate analysis (PCoA) of unweighted UniFrac phylogenetic distances.

Statistical analyses of the microbiota sequencing data were performed in R 3.0.2 [43]. The results on the community composition and the corresponding statistical analysis were based on relative abundances averaged from the sequencing replicates for each fraction (except for fraction 3 of the 2 h incubation, where only one sample was usable for sequencing). The differences in the mean relative abundance of bacterial taxa found in “heavy” and “medium” RNA-SIP fractions were assessed using one-way ANOVA. Differences in alpha diversity between “heavy” and “medium” fractions were analyzed using two-way ANOVA with time and density as factors. Differences with a *p* value < 0.05 were considered significant, while trends were defined as *p* > 0.05 but <0.10.

All sequencing data were submitted to GenBank and are publicly available under the accession number PRJNA340187.

### 2.5. Metabolic Profiling during Fermentation

Metabolite concentrations in the incubations were monitored using a high-performance liquid chromatography combustion isotope ratio mass spectrometer system (HPLC-C-IRMS) (Thermoquest, Bremen, Germany) as described previously [29, 44]. Concentrations and retention times of acetate, propionate, butyrate, and isobutyrate were determined by comparison with unlabeled standards. The isotopic signal of ^13^C/^12^C detected in the IRMS was calibrated with a CO_2_ gas standard, which was referenced against a methyl stearate working standard, which had been calibrated at the Max Planck Institute for Biogeochemistry, Jena, Germany (courtesy W.A. Brand).

## 3. Results and Discussion

### 3.1. Isolation, Amplification, and Sequencing of Isotope-Labeled 16S rRNA

**Figure 1:**
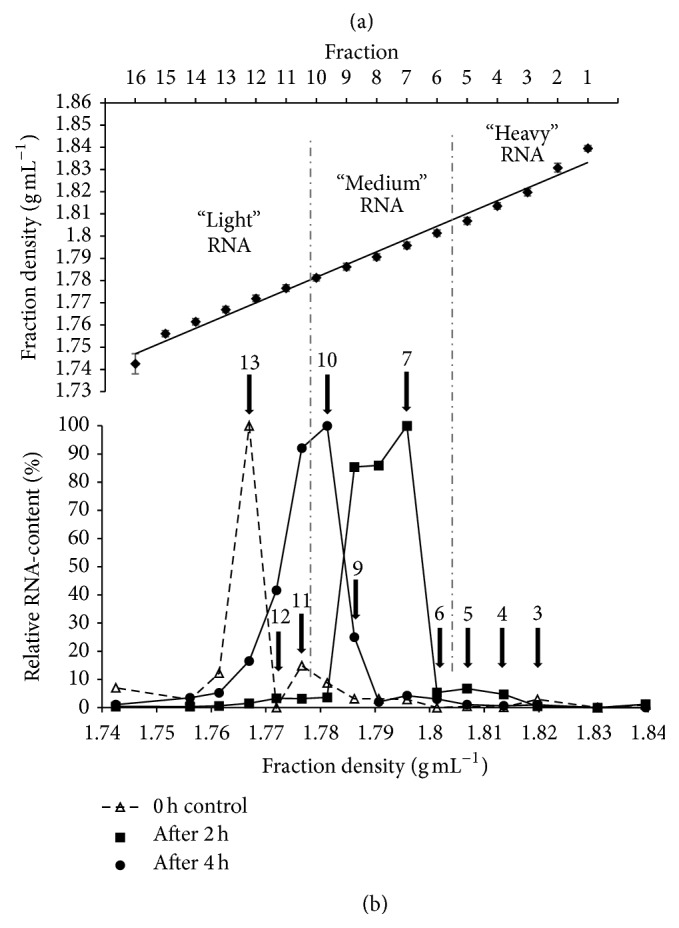
Density gradient formation and RNA distribution. (a) Buoyant densities (BD) of gradient fractions averaged over 12 gradients. The standard error of the mean (SEM) for each fraction was ≤0.0045 g mL^−1^. Vertical dash-dot lines classify the division of the gradients into “heavy” (fractions 1–5; BD 1.84–1.807 g mL^−1^), “medium” (fractions 6–10; BD 1.801–1.781 g mL^−1^), and “light” RNA fractions (fractions 11–16; BD 1.777–1.743 g mL^−1^). (b) Density-dependent RNA concentration in gradient fractions of the 40 mM [U^13^C]glucose cultures and the uncultured control. RNA was isolated from mice faeces at the start of the incubation (0 h control), after 2 h, and after 4 h from the [U^13^C] in vitro cultures, and resolved in a density gradient solution by ultracentrifugation. Separated RNA was harvested and quantified with a RiboGreen low range assay. To facilitate comparison between the gradients, the RNA content is given in relative units (%; fraction with the highest RNA concentration per gradient was set as 100%) [23]. Arrows indicate the gradient fractions, which were chosen for further downstream analysis by NGS.

The average density of the centrifugation gradients ranged from 1.743 g mL^−1^ (fraction 16) to 1.84 g mL^−1^ (fraction 1), which is in line with previous RNA-SIP studies and indicated an adequate density spectrum for efficient separation of isotope-labeled RNA [21–23, 25, 26]. Furthermore, the linear trend of the density spectrum indicated an appropriate gradient formation (Figure 1(a)). Based on the density, the gradients were further divided into “heavy” (BD 1.84–1.807 g mL^−1^; fractions 1–5), “medium” (BD 1.801–1.781 g mL^−1^; fractions 6–10), and “light” fractions (BD 1.777–1.743 g mL^−1^; fractions 11–16) (Figure 1).

While a noticeable amount of unlabeled control RNA (0 h) could be measured at averaged buoyant density (BD) between 1.796 g mL^−1^ (fraction 7) and 1.761 g mL^−1^ (fraction 14), the bulk of it accumulated in fraction 13, showing a BD of 1.767 g mL^−1^ (Figure 1(b)). After 2 h and 4 h of incubation with [U^13^C]glucose, RNA could be detected at BD ranging from 1.814 g mL^−1^ (fraction 4) to 1.761 g mL^−1^ (fraction 14) (Figure 1(b)). The observed shift of RNA towards higher densities indicated a substantial incorporation of ^13^C-label into the RNA of glucose-utilizing bacteria already after 2 h of incubation. With increased incubation time, the peak concentration of labeled RNA shifted back from fraction 7 (1.796 g mL^−1^) to fraction 10 (1.781 g mL^−1^) (Figure 1(b)), indicating a loss of label from the RNA.

The RNA distribution patterns throughout the different density fractions can be explained by glucose being rapidly fermented by many bacteria in the metabolically active community, leading to label incorporation into the RNA of all active bacteria present in the system. As a result, the ^13^C label could be found in a diverse range of RNA species at differing rates of incorporation, leading to accumulation of labeled RNA across a broad peak, designated as “medium” fractions at 2 h. Over the course of incubation, the label appeared to be diluted through replacement by unlabeled carbon molecules present in the system, most probably as a result of general RNA turnover [24, 45], leading to a shift in the peak RNA concentrations towards lower density fractions. Additionally, the broad spectrum of other available, unlabeled carbon sources stemming from carbohydrates and proteins in the faecal material [18], the large number of active bacteria present in the system and the general nature of glucose as a simple sugar probably resulted in further dilution of the isotope-label [23]. To ensure a significant and detectable ^13^C incorporation into the RNA, incubations in this study were conducted in minimal medium with 40 mM [U^13^C]glucose. Glucose concentrations in a double digit mM range can still be considered physiological for intestinal environments [23, 46]. However, in a previous RNA-SIP study within an intestinal environment, glucose concentrations < 40 mM did not lead to the production of sufficient labeled RNA for downstream analysis [23]. For more complex sugars, such as prebiotic carbohydrates, lower substrate concentrations might probably be sufficient to recover enough ^13^C-labeled RNA, as a more specific utilization process by a limited number of microbial populations can be assumed [23].

**Figure 2:**
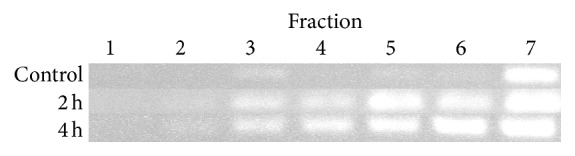
PCR analysis of selected gradient fractions. PCR amplification of cDNA reverse-transcribed from bacterial 16S rRNA harvested from gradient fractions 1–7 in a PCR assay resulted in an ~550 bp fragment with increased amounts of amplicons in “heavy” RNA-SIP fractions for the 2 h and 4 h incubations with [U^13^C]glucose. The fractions covered a BD spectrum from 1.84 g mL^−1^(fraction 1) to 1.796 g mL^−1^ (fraction 7). The picture is combined from three stained agarose gels after electrophoresis of the 16S RNA amplicons. For simplification, the size standard is not shown. All gels contained the same volume of PCR mixture. Faint bands in fractions 3 (uncultured control, 0 h) and 2 (2 h and 4 h ^13^C-cultures) represent low amounts of RNA amplicons which were not suitable for further analyses.

16S rRNA from cDNA was amplified from selected gradient fractions with universal bacterial primers. Amplification of cDNA obtained from incubations for 2 h and 4 h with [U^13^C]glucose yielded strong PCR products in “heavy” gradient fractions (fractions 3–5, BD 1.82–1.807 g mL^−1^, Figure 2), indicating an increased amount of ^13^C in the respective RNA. In contrast, amplicons from the unlabeled control cDNA (0 h) were obtained from fraction 7 (BD 1.796 g mL^−1^, Figure 2) to peak fraction 13 (BD 1.767 g mL^−1^).

In order to identify the most active bacterial populations involved in glucose assimilation, fractions for sequencing-based community analyses were chosen based on the density-dependent distribution of RNA in the gradients. “Heavy” fractions (fractions 3–5 of the 2 h and 4 h incubations) contained a low but still PCR-detectable amount of isotope-labeled RNA and hence phylogenetic information about the most prolific glucose utilizer in the system. To identify these particularly active community members, the community structure in the “heavy” fractions was compared to the community structure of fractions showing peak concentrations of bacterial RNA, that is, fractions 6 and 7 for the 2 h incubations, and fractions 9 and 10 for the 4 h incubations, representing the majority of bacteria that became only slightly labeled by the added [U^13^C]glucose, that is, bacteria which were less active glucose assimilators.

### 3.2. Characterization of Metabolically Active Populations

Sequencing and quality-trimming of the processed RNA samples yielded a total of 75,389 reads, with a maximum of 6,703 and a minimum of 1,008 reads per sample. The obtained sequences were affiliated with eight phyla, 16 classes, 24 orders, 42 families, and 50 genera over all analyzed fractions.

A depth of coverage of about 1000 sequences per samples is suggested to deduce the prevalence of species at 1% abundance with good accuracy [47]. Moreover, the aim of RNA-SIP studies is not to unravel the overall (and rare) diversity of an investigated habitat, but to identify microorganisms that have assimilated a labeled substrate by screening for differences in the relative abundances of microbial groups between isotope-labeled and unlabeled (or less labeled) RNA fractions. Clearly, results from our study showed the sequencing depth we obtained was sufficient to do so. Nevertheless, a higher sequencing depth might have yielded additional sequences of (rare) bacteria, which might have been involved in glucose assimilation, too.

**Figure 3:**
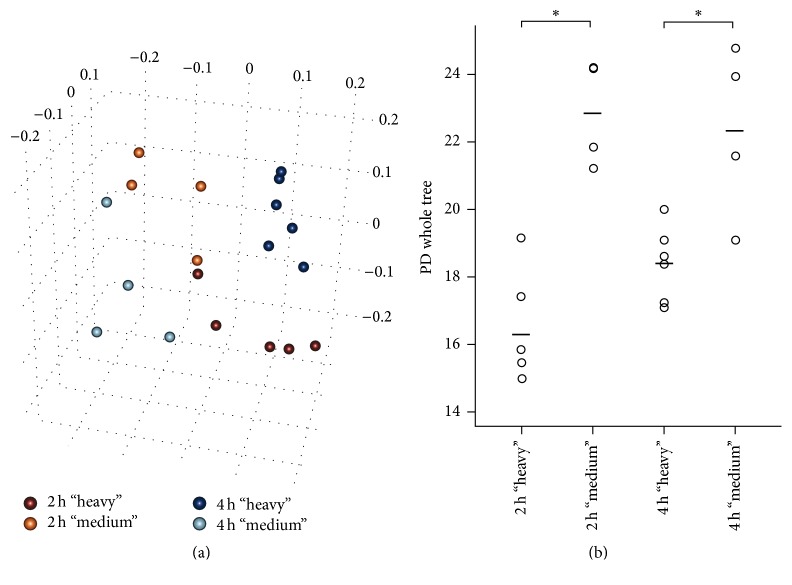
Bacterial diversity in the faecal microbiota. (a) Principal coordinate analysis (PCoA) of unweighted UniFrac phylogenetic distances of mouse faecal microbiota based upon different density RNA-SIP fractions. “Heavy” RNA-SIP fractions in comparison with the “medium” majority of the bacterial community of the 2 h and 4 h incubations in the presence of [U^13^C]glucose are shown. Each fraction was sequenced in duplicate (except fraction 3 of the 2 h incubation, where only one sample was usable for sequencing) and is represented as an individual point. (b) Faith's phylogenetic diversity estimate of “heavy” RNA-SIP fractions and the “medium” majority of the community of the 2 h and 4 h incubations, respectively. Each fraction was sequenced in duplicate (except fraction 3 of the 2 h incubation, where only one sample was usable for sequencing) and is displayed as an individual data point. Lines indicate the mean across the sequencing replicates and the designated different density fractions. *∗* indicates significant difference (*p* < 0.01) in complexity determined by two-way ANOVA.

**Figure 4:**
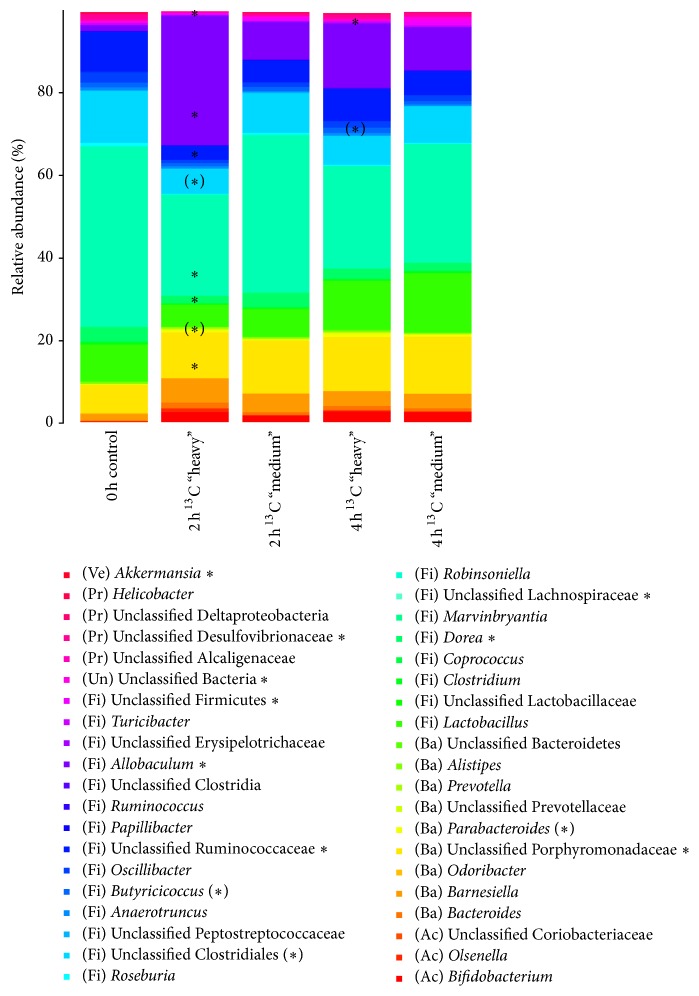
Relative abundance of bacterial taxa in different density RNA-SIP fractions. Stacked barplots showing the average community composition of different density RNA-SIP fractions from the uncultured control (0 h control) and from the 2 h and 4 h incubations in minimal medium with 40 mM [U^13^C]glucose. Shown are the 40 taxa with the highest mean relative abundance across all samples. *∗* indicates abundant taxa that are significantly different (*p* < 0.05) or are tending to differ (*∗*) (*p* < 0.10) in relative abundances in “heavy” fractions compared to their respective “medium” fractions determined by one-way ANOVA.

The metabolically active bacterial populations were characterized by comparing the community structure in the “heavy” fractions of the ^13^C labeled samples to the majority of the community, which accumulated in the “medium” fractions. When applying unweighted UniFrac PCoA analysis, a distinct clustering of “heavy” fractions from the “medium” majority of the community and between the 2 h and 4 h incubations with [U^13^C]glucose was observed (Figure 3(a)). Analysis of the mean bacterial community composition in the “heavy” fractions of both the 2 h and 4 h incubations revealed a complex community structure consisting of many bacterial taxa (Figure 4), but which still had a significantly lower diversity (*p* < 0.01) than the bacterial community in the “medium” fractions (Figure 3(b)). Furthermore, a comparison between the “heavy” fractions of the 2 h and 4 h incubations revealed that the bacterial diversity in these fractions increased with incubation time (Figure 3(b)). Differential clustering of samples among the different density RNA-SIP fractions (Figure 3(a)) suggests a strong relation in qualitative terms [48]. Because of the readily fermentable nature of glucose, a broad majority of the metabolically active bacterial community probably metabolized it quickly within 2 h of incubation and sequestered the isotope-label into their RNA. However, the observed clear phylogenetic delineation of “heavy” fractions from the “medium” majority of the metabolically active RNA species (Figure 3(a)) clearly showed that a distinct subset of the bacterial population was able to use the glucose more rapidly, while the remaining bacteria used the glucose less effectively or obtained the ^13^C label by “cross-feeding” on the glucose utilizers or their metabolic by-products, indicated by the tendency of separated cluster formation of the “heavy” fractions between the 2 h and 4 h incubations (Figure 3(a)). This finding is further corroborated by a significant lower diversity found in the “heavy” RNA-SIP fractions compared to the diversity represented by the “medium” majority of the community (Figure 3(b)). Moreover, the increasing diversity in the “heavy” fractions after 4 h of incubation (Figure 3(b)) suggests that more species were able to use the isotope-labeled glucose and/or its metabolites over time.

The microbial community composition before the incubation with glucose (0 h control) was analyzed from three “light” RNA fractions where most RNA accumulated. This microbial community in the control sample could be assigned to the Firmicutes (87.3%), Bacteroidetes (9.6%), and Proteobacteria (2.2%), representing the most abundant phyla. The most dominant taxa (≥1% relative abundance) within the Firmicutes, classified to the most detailed taxonomic level available, were unclassified Lachnospiraceae (43.8%), unclassified Clostridiales (12.6%), unclassified Ruminococcaceae (9.8%),* Lactobacillus* (9%),* Dorea *(3.4%),* Oscillibacter *(2.6%),* Allobaculum *(1.4%), and* Butyricicoccus* (1.2%). Among the Bacteroidetes, unclassified Porphyromonadaceae (6.9%) and* Barnesiella* (1.6%) represented the largest groups. Unclassified Desulfovibrionaceae (1.3%) were the largest group within the Proteobacteria. The phyla Actinobacteria, Tenericutes, Verrucomicrobia, and TM7 showed a low relative abundance (<1%). A small proportion (0.5%) of the total community was defined as unclassified Bacteria (Figure 4). The dominance of the phyla Firmicutes, Bacteroidetes, and to a lesser extent Proteobacteria is in good agreement with community compositions reported in other mice faecal 16S rRNA sequencing studies [49–53].

### 3.3. Identification of the Most Metabolically Active Intestinal Bacteria

The most metabolically active bacterial populations were determined by comparing the relative abundance of individual taxa in the “heavy” fractions of the ^13^C-labeled sample to that in the “medium” fractions. After 2 h of incubation with [U^13^C]glucose, the bacterial genus which differed most prominently between the different density fractions was* Allobaculum*, which was over 3-fold more abundant in the “heavy” RNA-SIP fractions than in the “medium” fractions (Table 1). Among the less abundant taxa,* Parabacteroides *showed a tendency to accumulate in the “heavy” fractions (Table 1). The observed increases were linked to a significant drop (*p* < 0.05) in proportions of the abundant unclassified Lachnospiraceae, unclassified Porphyromonadaceae, unclassified Ruminococcaceae,* Dorea,* and unclassified Desulfovibrionaceae, compared with their relative abundance in the “medium” fractions (Table 1). The unclassified Clostridiales tended to be found (*p* < 0.1) in reduced numbers in the “heavy” fractions. The most significant decrease (*p* = 0.001) was observed for the genus* Akkermansia*. Approximately 10-fold fewer sequences of this genus were detected in “heavy” RNA-SIP fractions than in the “medium” counterpart fractions, although the overall relative abundance of this taxon was low (<1%).

However, after 4 h of incubation with [U^13^C]glucose, the diversity of bacteria relatively enriched in the “heavy” fraction was increased compared with the diversity after 2 h of incubation. The genus that showed the most prominent tendency to accumulate in “heavy” fractions was* Butyricicoccus* (*p* < 0.1) (Table 1). Among the most abundant taxa,* Allobaculum*, unclassified Ruminococcaceae, and to a lesser extent* Dorea* were found in higher numbers in “heavy” fractions, which showed that the unclassified Ruminococcaceae and* Dorea* increased their representation in the “heavy” fractions from that at 2 h [U^13^C]-incubations (Table 1). Noticeably,* Akkermansia* was also found with an almost 4-fold increase in proportion in “heavy” fractions at 4 h compared with 2 h (Table 1). The global increases observed in the “heavy” RNA-SIP fractions corresponded to a significant proportional reduction of unclassified Firmicutes, unclassified Bacteria, and unclassified Bifidobacteriaceae (*p* < 0.01) (Table 1). Among the most abundant taxa,* Lactobacillus,* unclassified Lachnospiraceae, and unclassified Clostridiales showed reduced proportions.

The proportion of* Allobaculum*, a genus of the Erysipelotrichi class, was significantly higher in the “heavy” RNA-SIP fractions, indicating that the members of this genus could benefit the most from the excess glucose in the system, indicated by rapid label incorporation into their RNA. Interestingly,* Allobaculum* has also been found in increased numbers in prebiotic (oligofructose) treated mice in combination with a HFD (high-fat diet) but was reduced in HFD treatment lacking the prebiotic [54]. Moreover, in a study by Turnbaugh and colleagues, an increased relative abundance of the Erysipelotrichi class (comprising organisms closely related to* Clostridium innocuum, Eubacterium dolichum, *and* Catenibacterium mitsuokai*) was linked to the consumption of a Western diet (high-fat/high sugar) in mice containing a humanized gut microbiota [55]. Phylogenetic analysis of* Allobaculum stercoricanis* revealed that this organism is nearest phylogenetically related to members of* Clostridium *rRNA cluster XVI [56], which also includes* Clostridium innocuum *and* Eubacterium dolichum *[57, 58]. Based on these findings, we hypothesize that* Allobaculum* species are optimized for energy harvesting, as we have shown that it consumes simple sugars faster, and its relatives are linked to an adiposity-favouring microbiota [55]. Admittedly, it cannot be fully excluded that some inherent features of the* Allobaculum* RNA might be responsible for its higher relative abundance in the “heavy” fractions. Therefore, future studies might include additional ^12^C-controls to substantiate this finding. Nevertheless,* Allobaculum* was the only genus which showed significantly (*p* < 0.05) increased relative abundances in the “heavy” fractions after 2 h of incubation, whereas several other related low GC Gram-positive bacteria, such as the abundant Lachnospiraceae [59, 60], showed significantly reduced amounts there. Therefore, we are confident that the significant enrichment of* Allobaculum* RNA in “heavy” fractions can be attributed to an isotopic enriched RNA rather than to its native RNA BD, which is based on the low GC-content of the DNA of 37.9 mol% [56].

The genus* Akkermansia *was significantly less represented in the “heavy” fractions, indicating less glucose consumption than that by the other bacteria present in the system. This is consistent with* Akkermansia muciniphila* being adapted to a very specific ecological niche and using a very specific energy source, that is, mucin [61]. Hence, it is reasonable to assume that* Akkermansia* was probably less competitive for glucose than many other bacteria present in the community. Interestingly, a recent study also showed that this genus grows relatively poorly on glucose [62].

### 3.4. ^13^C Metabolite Production

**Figure 5:**
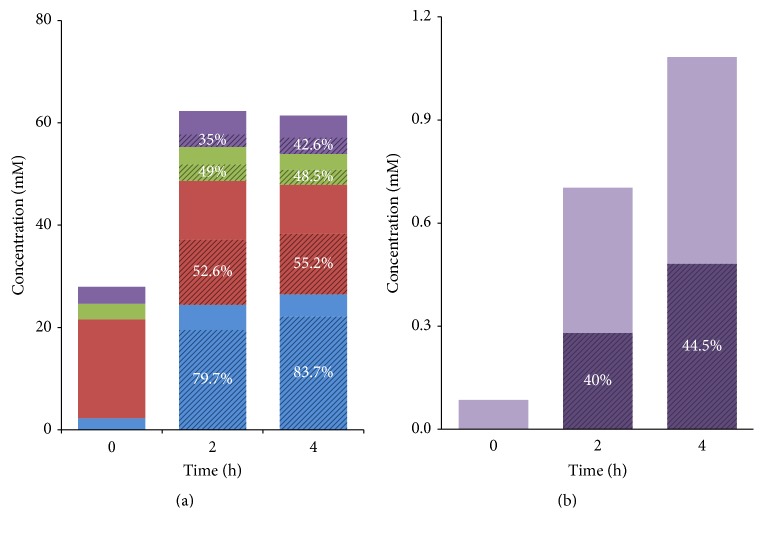
Time profiles of absolute concentrations and relative ^13^C-enrichments of the ^13^C-labeled metabolites after addition of [U^13^C]glucose (40 mM) at different time points. (a) Lactate (blue), acetate (red), propionate (green), butyrate (purple), and (b) isobutyrate concentrations. Solid colours show the absolute concentration measured in the samples. Stripes highlight the relative enrichment of ^13^C (presented as atom percent excess, APE) in the metabolites after labeling with [U^13^C]glucose, with percentages indicated. Average of duplicates is shown. % = atom%-excess (APE).

In the incubations with 40 mM [U^13^C]glucose, HPLC-IRMS analysis revealed ^13^C-labeled lactate, acetate, propionate, and butyrate as the main metabolic products; isotope-labeled isobutyrate was also measured, but in lower amounts (Figure 5). These products equated to 72.8% of the total added glucose carbon, while 47.4% of this recovered carbon was labeled with ^13^C. Lactate and SCFA which were detected already at the beginning of the incubations most probably stemmed from the inoculum, that is, the fresh faecal material. A more than 10-fold increase in total lactate concentration was determined after 2 h of incubation. Acetate, the most abundant SCFA present in human faeces [63, 64], only slightly increased during the course of incubations, whereas the concentrations of propionate and butyrate doubled (Figure 5(a)). Isobutyrate was detected only in low concentrations in fresh faecal slurry (0 h) (Figure 5(b)). After 2 h of incubation with [U^13^C]glucose, the ^13^C content of lactate, acetate, propionate, and butyrate reached 79.7, 52.6, 49, and 35 atom percent excess (APE) of the respective carbon-pools. These fractions remained approximately constant at 83.7, 55.2, 48.5 and 42.6 APE, after 4 h of incubation, respectively (Figure 5(a), stripes), suggesting that glucose was exhausted by 2 h and further fermentation was minimal. In the case of isobutyrate, ^13^C-enrichment increased to 40 APE and 44.5 APE after 2 h and 4 h of incubation in the presence of the isotope-label, respectively (Figure 5(b), stripes).

In this study, 40 mM of [U^13^C]glucose was rapidly fermented (within 2 h of incubation), yielding particularly lactate and acetate, and in addition to a lesser extent propionate and butyrate, as the main fermentation products. The profile of fermentation products corresponded to the composition of the microbial community. For example, many bacteria form lactic acid from glucose, including* Lactobacillus* spp., a well-known genus of the lactic acid bacteria [63, 65, 66], which occurred in relatively high abundances (up to 14.44%). The Bacteroidetes phylum was abundantly represented in the community by unclassified Porphyromonadaceae and* Barnesiella *representatives, which are associated with the production of acetate and propionate [66, 67]. Furthermore, several members of the abundant Lachnospiraceae family (*Clostridium *cluster XIVa) are known butyrate-producing species [59], and some of these have been linked to the conversion of lactate to butyrate [68]. The abundant genus* Allobaculum, *identified here as the most active glucose utilizer in this system, has been shown to yield mainly lactate and butyrate during glucose metabolism [56].

Our observation that significant proportions of the ^13^C label were found in lactate, acetate, propionate, and butyrate shows that the predominant carbon source used by the microbes was the isotope-labeled glucose. In particular, the high proportion of ^13^C label found in lactate (83.7 APE after 4 h) indicates that formation of lactate occurred predominantly from the [U^13^C]glucose (≥99 atom%) and only to a small extent from other unlabeled sources in the faecal material. Similarly, formation of acetate, propionate, and butyrate (all around 50 APE) occurred from glucose, albeit with a relatively larger contribution of other unlabeled sources. However, formation of these acids would also be conceivable from the labeled lactate [63, 68, 69], which was highly enriched with ^13^C. Moreover, more than 10-fold increase in produced lactate indicated a rapid fermentation and cycling of carbon through glycolysis [66, 70].

Interestingly, a loss of unlabeled acetate was observed during the course of incubation, since the total concentrations of acetate remained approximately constant, but became largely ^13^C-labeled (≥52.6 APE) with time. It might be speculated that conversion of acetate into butyrate via the* butyryl-CoA CoA-transferase *pathway occurred in the system [71]. This assumption is in line with the detection of increased, but still low, butyrate concentrations. In addition, acetate might have been converted to CO_2_ and CH_4_ by anaerobic microbial respiration (denitrification, desulfurication, and methanogenesis). However, we neither measured the respective metabolites nor specifically determined the relevant microbial populations in our samples to substantiate this hypothesis. Notably, Desulfovibrionales (comprising species capable of acetate-dependent desulfurication) were present in the samples investigated here [72], and acetoclastic archaea (*Methanosarcina *spp.) have been found in the faeces of herbivorous animals and ruminants [73, 74].

The branched-chain SCFA isobutyrate showed a greater than 10-fold increase in concentration at 4 h, indicating amino acid fermentation in the system [75, 76]. However, isotope enrichments of 44.5 APE were seen for isobutyrate at 4 h. Isobutyrate synthesis is formed from the degradation of valine [76]. Valine's original biosynthetic precursor is pyruvate. Highly ^13^C-enriched (>83.7 APE) pyruvate will occur in this system, as proven by the magnitude of ^13^C lactate (26.5 mM with 83.7 APE). Therefore, we estimate that the concentration of labeled valine arising from pyruvate is likely to be sufficient to provide for the small concentration (1.1 mM with 44.5 APE) isobutyrate measured. Furthermore, as there is no difference between the rates of valine degradation from within protein (albumin) or the free amino acid to isobutyrate by faecal microbiota in vitro [76], we hypothesize that the ^13^C-isobutyrate detected here may indicate “cross-feeding” upon valine or valine-containing proteins of [^13^C]glucose origin in this system.

In this study, we demonstrated that RNA-SIP in combination with metabolic profiling of ^13^C labeled fermentation products offers an efficient approach to link the identity of bacteria to their metabolic function and the metabolic by-products they produce. Insights into the metabolic activity of a complex microbial community of mice origin were gained using glucose as a model substrate. We have shown that a wide range of bacteria were active after glucose addition, which is not surprising as glucose is an easily fermentable energy source. Nevertheless, our data showed that distinct members of the faecal community were able to use the glucose more efficiently than others. Using mice as a model system is still considered as a powerful tool in microbiota research, as the microbiota can be investigated under controlled conditions (e.g., homogenous genetic background of the mice, consumed diet, and housing factors) [32]. Furthermore, the high similarity of the bacterial taxa and many anatomical similarities between mouse and the human digestive tract [32, 33] indicate that mice models are valid in human-associated microbiome studies. We will use the mouse system in future RNA-SIP studies to examine resistant starch utilization in vitro, followed by an in vivo feeding trial.

## Figures and Tables

**Table 1:** Summary of bacterial taxa differing between “heavy” and “medium” fractions of the 2 h and 4 h incubations with 40 mM [U^13^C]glucose. Shown are relative frequencies (mean ± SEM) in the “heavy” and “medium” fractions. Differences between the mean relative abundances found in these fractions were determined by one-way ANOVA. n.d. = not detected; Uc = unclassified.

Taxon	2 h ^13^C			4 h ^13^C		
	“Heavy” (%)	“Medium” (%)	*p* value	“Heavy” (%)	“Medium” (%)	*p* value
*Allobaculum*	31.39 ± 3.06	9.12 ± 3.6	0.02	15.58 ± 5.55	10.47 ± 4.46	0.56
UcLachnospiraceae	24.61 ± 2.75	38.31 ± 2.43	0.04	24.81 ± 1.2	28.82 ± 1.79	0.15
UcPorphyromonadaceae	11.07 ± 0.35	12.82 ± 0.07	0.03	13.14 ± 0.91	13.82 ± 0.42	0.61
UcClostridiales	6.04 ± 0.92	9.71 ± 1.27	0.095	7.06 ± 0.72	8.88 ± 1.46	0.29
UcRuminococcaceae	3.5 ± 0.2	5.33 ± 0.48	0.03	7.89 ± 0.74	5.82 ± 1.11	0.2
*Dorea*	1.75 ± 0.33	3.34 ± 0.03	0.03	2.42 ± 0.21	1.96 ± 0.47	0.37
*Parabacteroides*	0.63 ± 0.14	0.2 ± 0.03	0.099	0.89 ± 0.17	0.47 ± 0.12	0.17
UcDesulfovibrionaceae	0.28 ± 0.06	0.64 ± 0.09	0.04	1.06 ± 0.34	0.93 ± 0.19	0.79
*Akkermansia*	0.02 ± 0.01	0.21 ± 0.002	0.001	0.05 ± 0.03	0.01 ± 0.01	0.36
UcFirmicutes	0.23 ± 0.14	0.54 ± 0.01	0.17	0.1 ± 0.03	1.05 ± 0.11	0.002
UcBifidobacteriaceae	n.d.	0.05 ± 0.03	—	0.01 ± 0.01	0.06 ± 0.003	0.01
UcBacteria	0.16 ± 0.03	0.52 ± 0.18	0.08	0.13 ± 0.02	0.87 ± 0.17	0.01
*Butyricicoccus*	0.85 ± 0.15	0.98 ± 0.1	0.58	1.3 ± 0.18	0.7 ± 0.08	0.09
*Lactobacillus*	5.38 ± 0.52	6.73 ± 0.43	0.17	11.96 ± 1.96	14.44 ± 0.73	0.41
